# In-hospital mortality and one-year survival of critically ill patients with cancer colonized or not with carbapenem-resistant gram-negative bacteria or vancomycin-resistant enterococci: an observational study

**DOI:** 10.1186/s13756-023-01214-2

**Published:** 2023-02-08

**Authors:** Antonio Paulo Nassar Junior, Giulia Medola Del Missier, Ana Paula Agnolon Praça, Ivan Leonardo Avelino França e Silva, Pedro Caruso

**Affiliations:** 1 Intensive Care Unit, AC Camargo Cancer Center, São Paulo, Brazil; 2 Hospital Israelita Albert Einstein, São Paulo, Brazil; 3 Universidade Federal de São Paulo (UNIFESP), São Paulo, Brazil; 4 Department of Infectious Diseaes, AC Camargo Cancer Center, São Paulo, Brazil; 5 Pulmonary Division, Faculdade de Medicina da Universidade de São Paulo, Hospital das Clínicas, Heart Institute (InCor), São Paulo, Brazil

**Keywords:** Drug resistance, Multiple, Neoplasms, Intensive care units, Vancomycin-resistant enterococci, Carbapenem-resistant gram-negative bacteria, Hospital mortality, Survival analysis

## Abstract

**Background:**

Patients with cancer are at risk of multidrug-resistant bacteria colonization, but association of colonization with in-hospital mortality and one-year survival has not been established in critically ill patients with cancer.

**Methods:**

Using logistic and Cox-regression analyses adjusted for confounders, in adult patients admitted at intensive care unit (ICU) with active cancer, we evaluate the association of colonization by carbapenem-resistant Gram-negative bacteria or vancomycin-resistant enterococci with in-hospital mortality and one-year survival.

**Results:**

We included 714 patients and among them 140 were colonized (19.6%). Colonized patients more frequently came from ward, had longer hospital length of stay before ICU admission, had unplanned ICU admission, had worse performance status, higher predicted mortality upon ICU admission, and more hematological malignancies than patients without colonization. None of the patients presented conversion of colonization to infection by the same bacteria during hospital stay, but 20.7% presented conversion to infection after hospital discharge. Colonized patients had a higher in-hospital mortality compared to patients without colonization (44.3 vs. 33.4%; *p* < 0.01), but adjusting for confounders, colonization was not associated with in-hospital mortality [Odds ratio = 1.03 (0.77–1.99)]. Additionally, adjusting for confounders, colonization was not associated with one-year survival [Hazard ratio = 1.10 (0.87–1.40)].

**Conclusions:**

Adult critically ill patients with active cancer and colonized by carbapenem-resistant Gram-negative bacteria or vancomycin-resistant enterococci active cancer have a worse health status compared to patients without colonization. However, adjusting for confounders, colonization by carbapenem-resistant Gram-negative bacteria or vancomycin-resistant enterococci are not associated with in-hospital mortality and one-year survival.

**Supplementary Information:**

The online version contains supplementary material available at 10.1186/s13756-023-01214-2.

## Background

Multidrug-resistance bacteria (MDRB) are a leading health public threat, with millions of deaths annually attributed to MDRB infection [1]. MDRB colonization is the main risk factor for progression to future MDRB infection [2, 3].

Patients with cancer are considered a population at risk of MDRB colonization because prior hospitalization and antimicrobial treatment are risk factors for colonization [4], and these factors are frequently presented in patients with cancer [5]. Colonization by carbapenem-resistant Gram-negative (CR-GrN) bacteria and vancomycin-resistant enterococci (VRE) are particularly worrisome because these bacteria are associated with worse outcomes compared to other MDRB, such as extended spectrum beta-lactamase producing enterobacteria [6].

Patients with cancer are a growing population in intensive care units (ICU), making up approximately 30% of the ICU occupancy [7, 8]. The association of MDRB colonization with increased in-hospital mortality has been established in general populations of critically ill patients [9–11], but not in critically ill patients with cancer. Moreover, the association of MDRB colonization with one-year survival is unknown in patients with cancer. If MDRB colonization is associated with worse in-hospital outcomes or lower one-year survival, strategies for decolonization of selected patients, close monitoring or antibiotic stewardship have the potential for improve in-hospital outcomes and increase long-term survival.

In patients with active cancer admitted at ICU, we evaluated the association of colonization by CR-GrN bacteria or VRE with in-hospital mortality. We also evaluated the association of colonization with one-year survival.

## Methods

We designed a retrospective study including patients from the five ICUs of the AC Camargo Cancer Center (São Paulo–Brazil).

### Patients

For epidemiological surveillance, at the beginning of May 2017, the Hospital Infection and Prevention Control Department ordered a once-a-week rectal swab for routine screening of CR-GrN bacteria and VRE. Briefly, two separate rectal swabs were collected. For VRE detection, one swab was plated on ChromID^®^ VRE (bioMérieux, Brazil). For carbapenem-resistant enterobacterales and acinetobacter detection, the other swab was plated on CHROMagar mSuperCarba (Plastlabor, Brazil). Both plates were incubated in air at 35 ± 1 °C for a maximum of 48 h. If colonies were observed, identification was carried out using the Microflex mass spectrometry system (Bruker, Germany) (further details on Additional file 1).

Every Tuesday, all patients admitted for more than 48 h in the ICU were screened, excluding patients at end-of-life care. There was no routine screening for MDRB colonization outside ICU.

Between May 2017 and May 2019, we included all adult patients (≥ 18 years old), with active solid tumors or hematologic malignancies screened for CR-GrN bacteria and VRE during ICU stay. If a patient had multiple ICU admissions, only the first was considered. However, if a patient had multiple ICU admissions during the same hospital admission, and not the first, but a subsequent admission revealed CR-GrN bacteria or VRE colonization, that was the admission included in the study.

### Data collection

Upon ICU admission, patient’s age and sex, days in hospital before ICU admission, Simplified Acute Physiology Score (SAPS 3) [12]; Eastern Cooperative Oncology Group (ECOG) performance status [13]; type of cancer (nonmetastatic or metastatic solid tumors, hematologic malignancies); ICU architectural design (single-bed room or multibed room), type of ICU admission (planned or unplanned); and source of ICU admission (emergency room, ward or operating room) were recorded.

We recorded the result of the rectal swab as follow: negative, positive for CR-GrN bacteria, positive for VRE; or positive for both bacteria. We also recorded the ICU and hospital length of stay.

In patients colonized by CR-GrN bacteria or VRE, we evaluated the conversion of colonization to clinical infection by the same bacteria up until one-year after hospital discharge. The conversion was confirmed when the same CR-GrN bacteria or VRE initially identified in the rectal swab and with the same antibiotic-resistance profile was isolated in any culture and the antibiotic regimen was initiated or changed accordingly. The conversion detection was performed by two investigators that independently examined the electronic health record and microbiological laboratory results.

The main outcomes of the study were in-hospital mortality and one-year survival. There were not missing values.

### Data analysis

Categorical and continuous data were presented as absolute values (percentages) and median (25–75% interquartile range), respectively. Categorical variables were compared using the Chi-square test or Fisher’s exact test, as appropriate. Continuous variables were compared with the Mann–Whitney test.

### In-hospital mortality

A logistic regression model was used to evaluate the association of colonization with in-hospital mortality. We used directed acyclic graph to identify confounders [14]. We identified the following confounders: age, performance status, SAPS 3 score, type of ICU admission and type of cancer (Additional file 1: Fig. S1).

Odds ratio (OR) with 95% confidence interval was used to measure the association of colonization with in-hospital mortality. We presented the unadjusted and adjusted odds ratios.

### One-year survival

We used the multivariate Cox proportional hazards regression to compare the survival curve adjusted for confounders identified using directed acyclic graph. We identified the following confounders: age, performance status, and type of cancer (Additional file 1: Fig. S2). The time zero of the one-year follow-up period was the day of rectal swab screening.

Hazard ratio (HR) with 95% confidence interval was used to measure the association of colonization with one-year survival. We presented the unadjusted and adjusted hazard ratios.

Statistical analyses were performed using SPSS software (Version 23.0. Armonk, NY: IBM Corp). The directed acyclic graphs were created using the browser-based environment DAGitty [15]. We followed the recommendations of the STROBE (Strengthening the Reporting of Observational Studies in Epidemiology). statement that guides the report of observational studies [16] and the guidance for control of confounding and reporting of results in causal inference studies from editors of respiratory, sleep, and critical care journals [14]. *P* values ≤ 0.05 were considered significant.

## Results

**Fig. 1:**
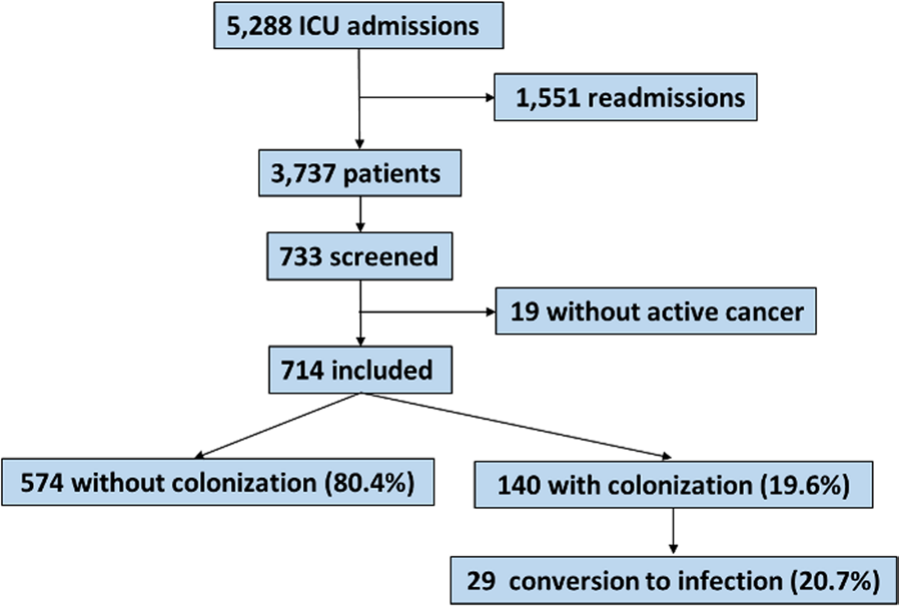
Flowchart of the study

3737 patients were admitted during the study period, 733 (19.6%) were elected to colonization screening and 714 patients were included in the study (Fig. 1). Only two patients had their second ICU admission included in the analyses and not the first admission. Both patients had a first negative rectal swab at first ICU admission, but few days later were readmitted at ICU and had a positive rectal swab.

One hundred and forty patients (19.6%) were colonized. Fifty patients (35.7%) were colonized by VRE, 73 (52.1%) by CR-GrN bacteria and 17 (12.2%) by both bacteria.

Upon ICU admission, patients with colonization more frequently came from ward, had longer hospital length of stay before ICU admission had unplanned ICU admission, worse performance status, higher predicted mortality upon ICU admission (SAPS3), and more hematological malignancies compared to patients without colonization (Table 1).

**Table 1:** Characteristics upon ICU admission of patients with cancer colonized by carbapenem-resistant Gram-negative bacteria, vancomycin-resistant enterococci or both

Variable	Total (n = 714)	Without colonization (n = 574)	With colonization (n = 140)	*P*
Age (years)	64 (53–73)	64 (53–73)	66 (54–74)	0.71
Male	389 (54.5%)	79 (56.4%)	310 (54.0%)	0.61
Type ICU admission				< 0.01
Planned	201 (28.2%)	182 (31.7%)	19 (13.6%)	
Unplanned	513 (71.8%)	392 (68.3%)	121 (86.4%)	
Before ICU admission				< 0.01
Operating room	243 (34.0%)	214 (37.3%)	29 (20.7%)	
Emergency room	181 (25.4%)	151 (26.3%)	30 (21.4%)	
Ward	290 (40.6%)	209 (36.4%)	81 (57.9%)	
ICU design				0.51
Single-bed room	316 (44.3%)	258 (44.9%)	58 (41.4%)	
Multibed room	398 (55.7%)	316 (55.1%)	82 (58.6%)	
Performance status (ECOG)				< 0.01
0–1	409 (57.3%)	353 (61.5%)	56 (40.0%)	
2	159 (22.3%)	122 (21.3%)	37 (26.4%)	
3–4	146 (20.4%)	99 (17.2%)	47 (33.6%)	
SAPS 3	63 (53–72)	62 (51–72)	68 (59–79)	< 0.01
Type of cancer				0.02
Solid tumor	623 (87.3%)	510 (88.9%)	113 (80.7%)	
Hematological	91 (12.7%)	64 (11.1%)	27 (19.3%)	
Metastatic tumor	277 (44.5%)	228 (44.8%)	49 (43.6%)	0.85
Days in hospital before ICU	1 (0–9)	1 (0–7)	3 (1–16)	< 0.01
Days in ICU before screening	3 (2–5)	3 (2–5)	3 (2–5)	0.98
ICU length of stay (days)	6 (4–10)	6 (4–9)	8 (5–12)	< 0.01
Hospital length of stay(days) (days)	22 (13–38)	21 (13–35)	28 (17–53)	< 0.01

Categorical data are presented as median (25–75% interquartile range). Continuous data were presented as absolute values (percentages)

*ICU* Intensive Care Unit, *planned ICU admission* postoperative elective surgery, *unplanned ICU admission* medical reason or postoperative emergency surgery, *ECOG* Eastern Cooperative Oncology Group, *SAPS3* Simplified Acute Physiology Score (SAPS 3), *Days in ICU before screening* ICU length of stay before colonization screening with rectal swabs

### In-hospital mortality

No one of the colonized patients presented conversion to infection during the hospital stay. Patients with colonization had a higher in-hospital mortality compared to patients without colonization (44.3 vs. 33.4%; *p* < 0.01) [unadjusted OR = 1.58 (1.09–2.30)]. However, after adjusting for age, performance status, SAPS 3 score, type of ICU admission and type of cancer, colonization was not associated with in-hospital mortality [adjusted OR = 1.03 (0.77–1.99)].

### One-year survival

Among the 140 colonized patients, 29 (20.7%) presented conversion of colonization to infection. The conversion rate to infection was 27.4% (20/73) for patients colonized by CR-GrN, 8.0% (4/50) for patients colonized by VRE, and 29.4% (5/17) for patients colonized by both bacteria. The most frequent infections sites were urinary tract (31.0%), respiratory tract (20.7%), intrabdominal (17.2%) and bacteremia (13.8%). The interval between colonization diagnosis and infection was 27 days (15–60). Upon ICU admission, the characteristics of the colonized patients that converted or not infection were similar (Table 2).

**Table 2:** Characteristics upon ICU admission of colonized patients with active cancer with or without conversion to infection

Variable	Total (n = 140)	Colonization without infection (n = 111)	Colonization with infection (n = 29)	*P*
Age (years)	66 (54–74)	67 (55–74)	61 (48–75)	0.21
Male	79 (56.4%)	63 (56.8%)	16 (55.2%)	0.99
Type ICU admission				0.23
Planned	19 (13.6%)	13 (11.7%)	6 (20.7%)	
Unplanned	121 (86.4%)	98 (88.3%)	23 (79.3%)	
Before ICU admission				0.08
Operating room	29 (20.7%)	19 (17.1%)	10 (34.5%)	
Emergency room	30 (21.4%)	23 (20.7%)	07 (24.1%)	
Ward	81 (57.9%)	69 (62.2%)	12 (41.4%)	
ICU design				0.68
Single-bed room	58 (41.4%)	45 (40.5%)	13 (44.8%)	
Multibed room	58 (41.4%)	45 (40.5%)	13 (44.8%)	
Performance status (ECOG)				0.75
0–1	56 (40.0%)	46 (41.4%)	10 (34.5%)	
2	37 (26.4%)	28 (25.2%)	9 (31.0%)	
3–4	47 (33.6%)	37 (33.3%)	10 (34.5%)	
SAPS 3	68 (56–79)	68 (58–80)	62 (50–72)	0.08
Type of cancer				0.99
Solid tumor	113 (80.7%)	89 (80.2%)	24 (82.8%)	
Hematological	27 (19.3%)	22 (19.8%)	05 (17.2%)	
Metastatic tumor	61 (43.6%)	46 (41.4%)	15 (51.7%)	0.40

Categorical data are presented as median (25–75% interquartile range). Continuous data were presented as absolute values (percentages)

*ICU* Intensive Care Unit, *planned ICU admission* postoperative elective surgery, *unplanned ICU admission* medical reason or postoperative emergency surgery, *ECOG* Eastern Cooperative Oncology Group, *SAPS3* Simplified Acute Physiology Score (SAPS 3)

**Fig. 2:**
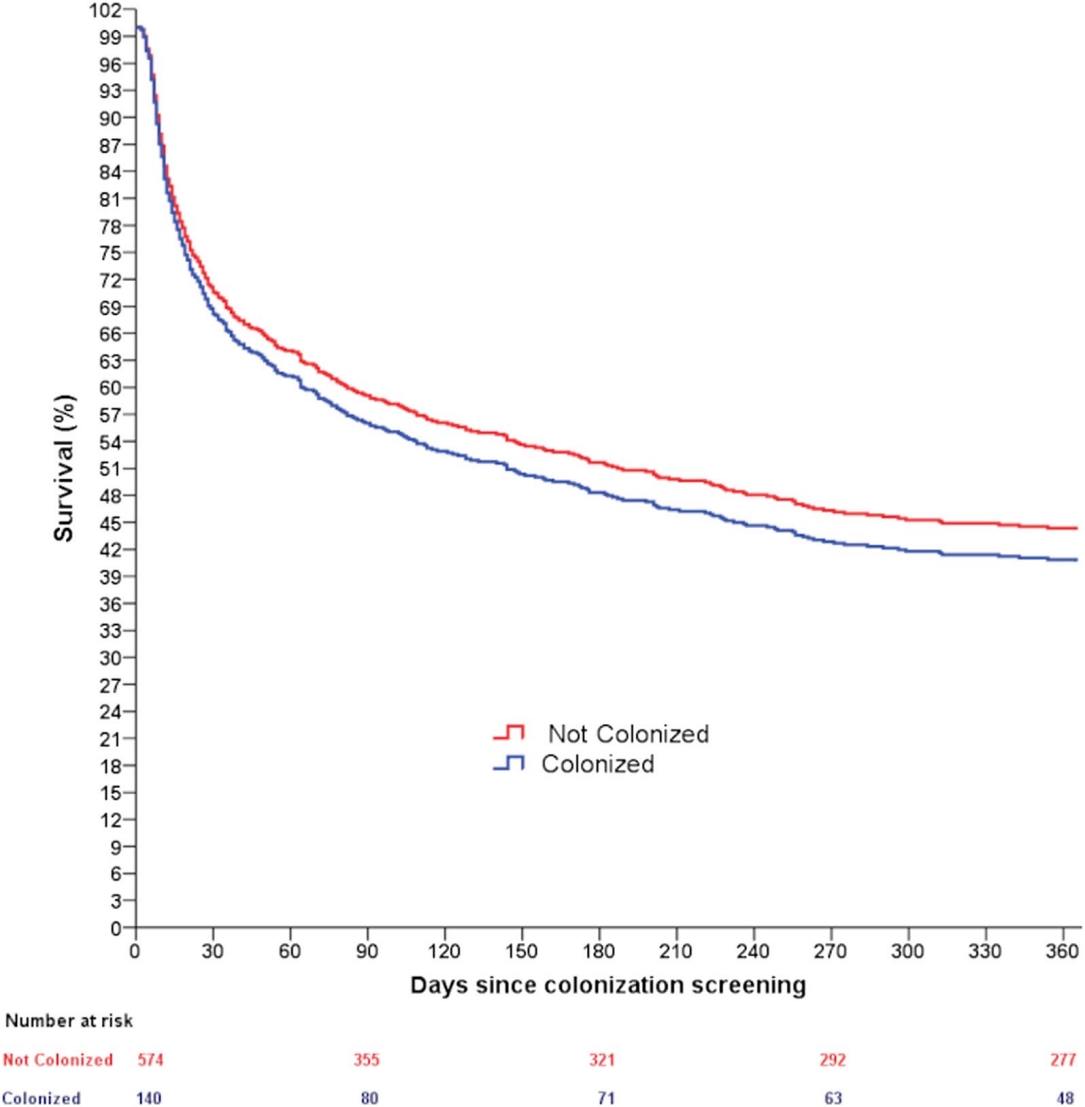
Survival curves of patients with (blue) or without (red) colonization by carbapenem-resistant Gram-negative bacteria or vancomycin-resistant enterococci

Two hundred and ninety-seven (51.7%) patients without colonization and 92 (65.7%) patients with colonization died during the one-year follow-up period [unadjusted HR = 1.38 (1.09–1.74)]. However, adjusting for confounders, colonization was not associated with lower one-year survival compared to patients without colonization [adjusted HR = 1.10 (0.87–1.40)] (Fig. 2).

## Discussion

In patients with active cancer admitted at ICU, we showed that colonization by CR-GrN bacteria or VRE was not associated with in-hospital mortality and one-year survival.

In our study, the prevalence of colonization among patients with cancer admitted at ICU was 19.6%. The comparison of MDRB prevalence among the studies is difficult because the studies evaluated different MDRB and populations. Evaluating a general critically ill patient population, Nseir et al. [4] showed a 13% prevalence of colonization or infection at ICU admission, while Masse et al. [3] showed that 34% of patients had colonization or infection during ICU stay. Among patients in hematological institutions, Cattaneo et al. [6] showed that 6.5% had colonization. Forcina et al. [17] showed that 16.9% of patients had colonization by multidrug-resistant Gram-negative bacteria before an allogenic hematopoietic stem cell transplantation. Although the comparison is troublesome, the MDRB prevalence in our study is of the same magnitude of other studies.

In our study, the in-hospital mortality of patients with colonization was higher compared to patients without colonization. However, the comparison is not straightforward because patients with colonization had worse performance status, longer hospital length of stay before ICU admission, more unplanned ICU admission, higher SAPS 3 score, and more hematological malignancies. All these variables have been strongly associated with higher in-hospital mortality [8, 18–20]. Adjusting for the identified confounders, colonization was not associated with in-hospital mortality.

Our results showed that the conversion to infection was higher among patients colonized by CR-GrN compared to patients colonized by VRE. Likewise, Cattaneo and colleagues showed a higher percentage of conversion to infection among patients colonized by CR-GrN compared to patients colonized by VRE. The authors evaluated patients with cancer and the conversion from colonization to bloodstream infection was 14.1% for patients colonized by CR-GrN bacteria compared to 11.1% for patients colonized by VRE [6]. Liss et al. [21] showed a conversion rate of 2.0% for patients with cancer colonized by VRE, while Girmenia et al. [22] showed that 25.8% of autologous stem cell transplant and 39.2% of allogenic stem cell transplant patients presented a conversion from CR-GrN colonization to infection. The difference in conversion rates probably reflects the variability in bacteria virulence and patients’ immunological competence.

Adjusting for confounders, we also showed that colonization was not associated with one-year survival, corroborating the knowledge that cancer-associated variables are more associated with long-term survival than other clinical variables [21] Upon ICU admission, the characteristics of the colonized patients that converted or not to infection over one-year was similar. Therefore, either the causes of conversion of colonization to infection were not recorded upon ICU admission or were present only after the colonization diagnosis.

We believe that our study has strengths worth mentioning. We evaluated a population that had not been previously evaluated, patients with cancer demanding ICU admission. We recorded short-term (in-hospital mortality) and long-term (one-year survival) outcomes of patients with or without CR-GrN or VRE colonization. One clinical implication of the present study is that patients with cancer and poor health status should be considered to MDRB screening. Another implication is that oncologists should not consider MDRB colonization in the short or long-term prognostication of patients with cancer. However, the impact of conversion to infection on short or long-term survival needs further investigation.

Our study has limitations that shall be mentioned. First, it was a single- center study and the results could be different in ICUs with different population and care. Our results are limited to patients colonized with CR-GrN or VRE. We did not record detailed information about the immunosuppression status, such as recent use of chemotherapy. However, active cancer is already considered immunosuppression. We only screened 19.6% of patients admitted to ICU because due to economical constraints, we decided to screen only on Tuesdays. The results could be different if we had screened all patients because patients with shorter stay had a lower chance of sampling than those with longer stay, resulting in overrepresentation of patients with longer stay. Finally, the conversion of colonization to infection was considered using phenotypically and not genotypically identical bacteria.

## Conclusions

In conclusion, adult critically ill patients with active cancer and colonized by carbapenem-resistant Gram-negative bacteria or vancomycin-resistant enterococci active cancer have a worse health status compared to patients without colonization. However, adjusting for confounders, colonization by carbapenem-resistant Gram-negative bacteria or vancomycin-resistant enterococci are not associated with in-hospital mortality and one-year survival.

## Supplementary Information

**Additional file 1.** Supplementary figures.

## Data Availability

The datasets generated during the current study are available from the corresponding author on reasonable request.

## Abbreviations

CR-GrN: Carbapenem-resistant gram-negative bacteria
ECOG: Eastern cooperative oncology group
HR: Hazard ratio
ICU: Intensive care unit
MDRB: Multidrug-resistance bacteria
OR: Odds ratio
SAPS 3: Simplified acute physiology score
STROBE: Strengthening the reporting of observational studies in epidemiology
VRE: Vancomycin-resistant enterococci
