# Supplementary material for: In-hospital mortality and one-year survival of critically ill patients with cancer colonized or not with carbapenem-resistant gram-negative bacteria or vancomycin-resistant enterococci: an observational study
Source: Antimicrob Resist Infect Control. 2023 Feb 8;12:8. doi: 10.1186/s13756-023-01214-2 (PMC9906932; doi:10.1186/s13756-023-01214-2)
Supplement: Supplementary file 1 — Additional file 1. Supplementary figures. [file 13756_2023_1214_MOESM1_ESM.docx]

**Additional File 1**

**Methods**

*Rectal swab method*

Two separate rectal swabs were collected and transported in Amies transport medium with charcoal (Copan, USA). For vancomycin-resistant enterococci detection, the first swab was plated on ChromID® VRE (bioMérieux, Brazil). For carbapenem-resistant enterobacterales and acinetobacter detection, the second swab was plated on CHROMagar mSuperCarba (Plastlabor, Brazil). Both plates were incubated in air at 35 ± 1°C for a maximum of 48 hours. If colonies were observed, identification was carried out using the Microflex mass spectrometry system (Bruker, Germany). Vancomycin or carbapenem resistance was confirmed by disk diffusion (EUCAST). Enterobacterales were also tested against meropenem (MEM) disks containing EDTA or phenylboronic acid (FBA). Isolates showing an inhibition zone diameter enhancement ≥ 5mm for MEM+EDTA were classified as metallo-β-lactamase (Ambler class B carbapenemase) producers. Those showing an inhibition zone diameter enhancement ≥ 5mm for MEM+FBA were classified as class A carbapenemase producers. Isolates with metallo-β-lactamase phenotype were also tested for bla NDM by PCR. MEM-resistant isolates showing no increase in zone diameter after EDTA or FBA addition were tested for bla NDM , bla KPC, and bla OXA-48 by PCR.


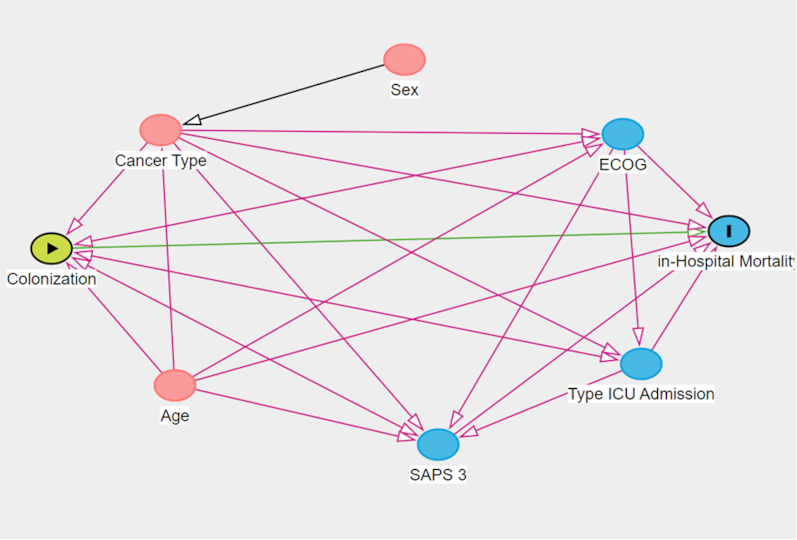


**Figure A1**. Directed acyclic graph used to identify confounders in the evaluation of the association of colonization with in-hospital mortality.


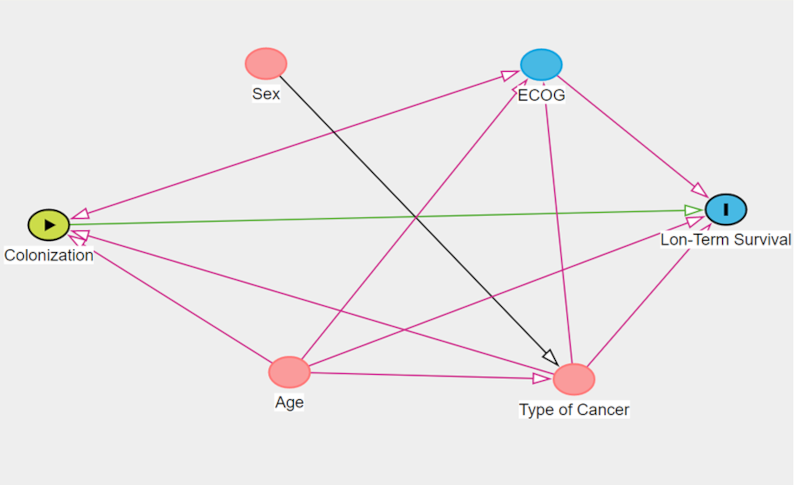


**Figure A2**. Directed acyclic graph used to identify confounders in the evaluation of colonization with one-year survival.
